# Influence of post-stroke fatigue on reaction times and corticospinal excitability during movement preparation

**DOI:** 10.1016/j.clinph.2020.11.012

**Published:** 2021-01

**Authors:** William De Doncker, Katlyn E. Brown, Annapoorna Kuppuswamy

**Affiliations:** a Department of Clinical and Movement Neuroscience, Institute of Neurology, University College London, UK; b University of Waterloo, Department of Kinesiology, Faculty of Applied Health Sciences, Waterloo, ON, Canada

**Keywords:** Stroke, Fatigue, TMS, Movement preparation, PSF, post-stroke fatigue, TMS, transcranial magnetic stimulation, M1, primary motor cortex, EMG, electromyography, RT, reaction time, HADS, hospital anxiety and depression scale, NHPT, nine-hole peg test, FDI, first dorsal interosseous, RMT, resting motor threshold, WS, warning stimulus, IS, imperative stimulus, WP, warning period, RT30, 30% Reaction time, RT50, 50% Reaction time, RT70, 70% Reaction Time, FSS-7, fatigue severity scale, SDMT, symbol digit modalities test

## Abstract

**Objectives:**

Reduced corticospinal excitability at rest is associated with post-stroke fatigue (PSF). However, it is not known if corticospinal excitability prior to a movement is also altered in fatigue which may then influence subsequent behaviour. We hypothesized that the levels of PSF can be explained by differences in modulation of corticospinal excitability during movement preparation.

**Methods:**

73 stroke survivors performed an auditory reaction time task. Corticospinal excitability was measured using transcranial magnetic stimulation. Fatigue was quantified using the fatigue severity scale. The effect of time and fatigue on corticospinal excitability and reaction time was analysed using a mixed effects model.

**Results:**

Those with greater levels of PSF showed reduced suppression of corticospinal excitability during movement preparation and increased facilitation immediately prior to movement onset (β = −0.0066, t = −2.22, p = 0.0263). Greater the fatigue, slower the reaction times the closer the stimulation time to movement onset (β = 0.0024, t = 2.47, p = 0.0159).

**Conclusions:**

Lack of pre-movement modulation of corticospinal excitability in high fatigue may indicate poor sensory processing supporting the sensory attenuation model of fatigue.

**Significance:**

We take a systems-based approach and investigate the motor system and its role in pathological fatigue allowing us to move towards gaining a mechanistic understanding of chronic pathological fatigue.

## Highlights

• Higher the fatigue, lesser the inhibition in movement preparation in stroke survivors.
• Higher the fatigue, lesser the pre-movement facilitation and slower the reaction times.
• Poor excitability modulation supports sensory attenuation model of fatigue.

## 1 Introduction

Post-stroke fatigue (PSF) is one of the most commonly self-reported symptoms after stroke that has significant implications for morbidity, disability, quality of life and mortality (Barbour and Mead 2012). The majority of stroke survivors report fatigue in the first few weeks after stroke. This is linked to high levels of inflammation immediately following an injury and such fatigue is part of sickness behaviour (Dantzer and Kelley 2007). The more debilitating symptom however, is fatigue that fails to resolve and persists for months or even years after the stroke. The reported incidence of PSF does not depend on stroke type, lesion location, age or gender (Ingles et al., 1999, Schepers et al., 2006). PSF has significant overlap with other affective symptoms such as depression, anxiety, pain and sleep disturbances, however medication targeting these other affective symptoms, such as antidepressants, have proven to be unsuccessful in treating fatigue (Karaiskos et al., 2012, Naess et al., 2012). Despite the high prevalence of PSF, an understanding of the underlying mechanisms that underpin PSF is currently lacking (De Doncker et al. 2018).

We have previously shown that self-selected ballistic movement speeds are slower and resting corticospinal excitability assessed using transcranial magnetic stimulation (TMS) is reduced in the affected hemisphere of stroke survivors who report high levels of PSF (Kuppuswamy et al., 2015a, Kuppuswamy et al., 2015b). Speed of ballistic movements is dependent on the ability of the motor cortex to activate necessary corticospinal output to initiate movement and are intrinsically linked to corticospinal excitability (Jäncke et al. 2004). The state of the motor cortex at a time prior to movement, commonly referred to as the ‘movement preparation’ period plays a crucial role in determining ballistic movement speeds. Notably, corticospinal excitability does not only change during movement initiation but also undergoes distinct modulation during movement preparation (Cisek and Kalaska 2010).

When preparing for a voluntary movement, there are substantial changes in the activity of neurons within the premotor and primary motor cortex (M1) despite no electromyographic (EMG) activity (Tanji and Evarts 1976). Movement preparation has been extensively studied in humans using TMS over M1 to probe corticospinal excitability changes during cue-driven reaction time (RT) paradigms (Hasbroucq et al., 1997, Duque and Ivry, 2009, Hannah et al., 2018, Ibáñez et al., 2020). Cues for guiding movement are probabilistic in nature and learning the probabilities of upcoming movements enable the motor system to prepare motor output prior to movement initiation. Suppression of corticospinal excitability prior to movement initiation is seen in muscles that are both involved and uninvolved in an action (Greenhouse et al., 2015, Bestmann and Duque, 2016, Duque et al., 2017). The predicted aspects of sensory information (target cues in RT paradigms) are represented explicitly in the modulation of corticospinal excitability during movement preparation. One of the proposals of sensory attenuation model of fatigue is that, the inability to suppress predicted sensory stimuli results in high perceived effort leading to fatigue (Kuppuswamy 2017). Therefore, pre-movement excitability representing predicted sensory information may be altered in those who exhibit high levels of fatigue. Indeed, such differences in corticospinal excitability during movement preparation have previously been reported in multiple sclerosis patients with high and low fatigue (Morgante et al. 2011). Behaviourally, such changes may result in reduced movement speeds (mediated by high perceived effort) which has been seen in stroke survivors with high fatigue (Kuppuswamy et al. 2015a). We also reported no differences in reaction time. In this study however, we choose to study premovement excitability in a reaction time task and not movement time for two reasons. One, it is known that the technique of TMS used to study premovement excitability induces a change in reaction time and it is not known if fatigue influences the TMS induced delay in reaction time. Secondly, reaction time and movement time are inextricably linked, and any influence on reaction time will subsequently influence movement time. Therefore, the aim of this study was to investigate the modulation of corticospinal excitability in a reaction time task in stroke survivors with varying severity of PSF.

## 2 Materials and methods

### 2.1 Subjects

This is a cross-sectional observational study approved by the London Bromley Research Ethics Committee (REC reference number: 16/LO/0714). Stroke survivors were recruited via the Clinical Research Network from the University College NHS Trust Hospital, a departmental Stroke Database and from the community. All stroke survivors were screened prior to the study based on the following criteria: (1) first-time ischaemic or haemorrhagic stroke; (2) stroke occurred at least 3 months prior to the study; (3) no other neurological disorder; (4) not taking anti-depressants or any other centrally acting medication; (5) not clinically depressed with depression scores ≤ 11 assessed using the Hospital Anxiety and Depression Scale (HADS); (6) no sensory impairment; (7) grip strength and manual dexterity of the affected hand (at least 60% of the unaffected hand) assessed using a hand-held dynamometer and the nine hole peg test (NHPT) respectively; (8) no contraindications to TMS. Seventy-three stroke survivors took part in the study (demographics shown in Table 1). All stroke survivors provided written informed consent in accordance with the Declaration of Helsinki.

**Table 1:** Demographics of all stroke survivors that took part in the study. Significance values for FSS-7 are reported for continuous and categorical data respectively. (NHPT = Nine Hole Peg Test; SDMT = Symbol Digit Modalities test; HADS = Hospital Anxiety and Depression Scale; RMT = resting motor threshold; MSO = maximum stimulator output).

	data (N = 73)	P-value
**FSS-7**		
Mean (SD)	3.69 (1.84)	
**Gender**		*P* = 0.5637
Females	24	
Males	49	
**Hemisphere Affected**		*P* = 1.0000
Left Hemisphere	40	
Right Hemisphere	33	
**Dominant Hand**		*P* = 0.3775
Right Hand	67	
Left Hand	6	
**Age (years)**		
Mean (SD)	61.55 (12.11)	*P* = 1.0000
**Grip (% unaffected hand)**		
Mean (SD)	91.49 (18.82)	*P* = 1.0000
**NHPT (% unaffected hand)**		
Mean (SD)	89.70 (23.66)	*P* = 1.0000
**SDMT**		
Mean (SD)	1.19 (0.43)	*P* = 1.0000
**Depression - HADS**		
Mean (SD)	4.56 (2.95)	*P* < 0.0001
**Anxiety - HADS**		
Mean (SD)	5.04 (3.86)	*P* = 0.0069
**RMT (% MSO)**		
Mean (SD)	47.81 (10.69)	*P* = 1.0000
**0.5 mV Intensity (% MSO)**		
Mean (SD)	59.03 (13.19)	*P* = 1.0000
**Reaction Time (s)**		
Mean (SD)	0.18 (0.06)	*P* = 0.3963

### 2.2 Surface electromyogram and transcranial magnetic stimulation

Recordings were carried out on the first dorsal interosseous (FDI) muscle of the affected hand. Following skin preparation using alcohol swabs, EMG recordings were obtained from the FDI muscle using surface neonatal prewired disposable electrodes (1041PTS Neonatal Electrode, Kendell) in a belly-tendon montage with the ground positioned over the flexor retinaculum of the hand. The signal was amplified with a gain of 1000 (D360, Digitmer, Welwyn Garden City, UK), bandpass filtered (100–1000 Hz), digitized at 5 kHz (Power1401, CED, Cambridge, UK) and recorded with Signal version 6.04 software (CED, Cambridge, UK). EMG recordings enabled the measurement of motor evoked potentials (MEPs).

A standard monophasic TMS device (Magstin 2002, Magstim, Whitland, Wales) connected to a figure-of-eight coil (wing diameter, 70 mm) was used to stimulate the hand area of M1 in the hemisphere affected by the stroke. The coil was held tangentially on the scalp at an angle of 45° to the mid-sagittal plane to induce a posterior-anterior current across the central sulcus. The subjects were instructed to stay relaxed with their eyes open and their legs uncrossed. The motor ‘hotspot’ of the FDI muscle was determined as follows: the vertex (cross-over point between the mid-point between the two tragi and midpoint between nasion and inion) was marked using a dry wipe marker. Four centimetres lateral and 2 cm anterior from the vertex was then marked on the contralateral hemisphere, which is the approximate location of M1. This was used as a rough guide for a starting point for determining the hotspot for the first dorsal interosseous. At 50% maximal stimulator output (MSO) (or higher or lower in some patients) the coil was moved in 1 cm blocks for ~2 cm anterior, posterior, lateral and medial to the marked region. Three stimuli were delivered at each spot and the location with the highest average motor evoked potential response was taken as the hotspot.

### 2.3 Resting motor threshold and 0.5 mV intensity

Resting motor threshold (RMT) was defined as the lowest intensity of stimulation (% MSO) required to evoke a peak-to-peak MEP amplitude at the hotspot of at least 50 μV in a minimum of 5 of 10 consecutive trials while subjects were at rest. Throughout the experiment, the stimulator setting was adjusted to produce a target MEP size of 0.5 mV. This was defined as the stimulator setting (determined to the nearest 1% of MSO) required to evoke a peak-to-peak MEP amplitude of ≥ 0.5 mV in a minimum of 5 of 10 consecutive trials. A 0.5 mV MEP was achieved in all patients.

### 2.4 Simple warned reaction time task

**Fig. 1:**
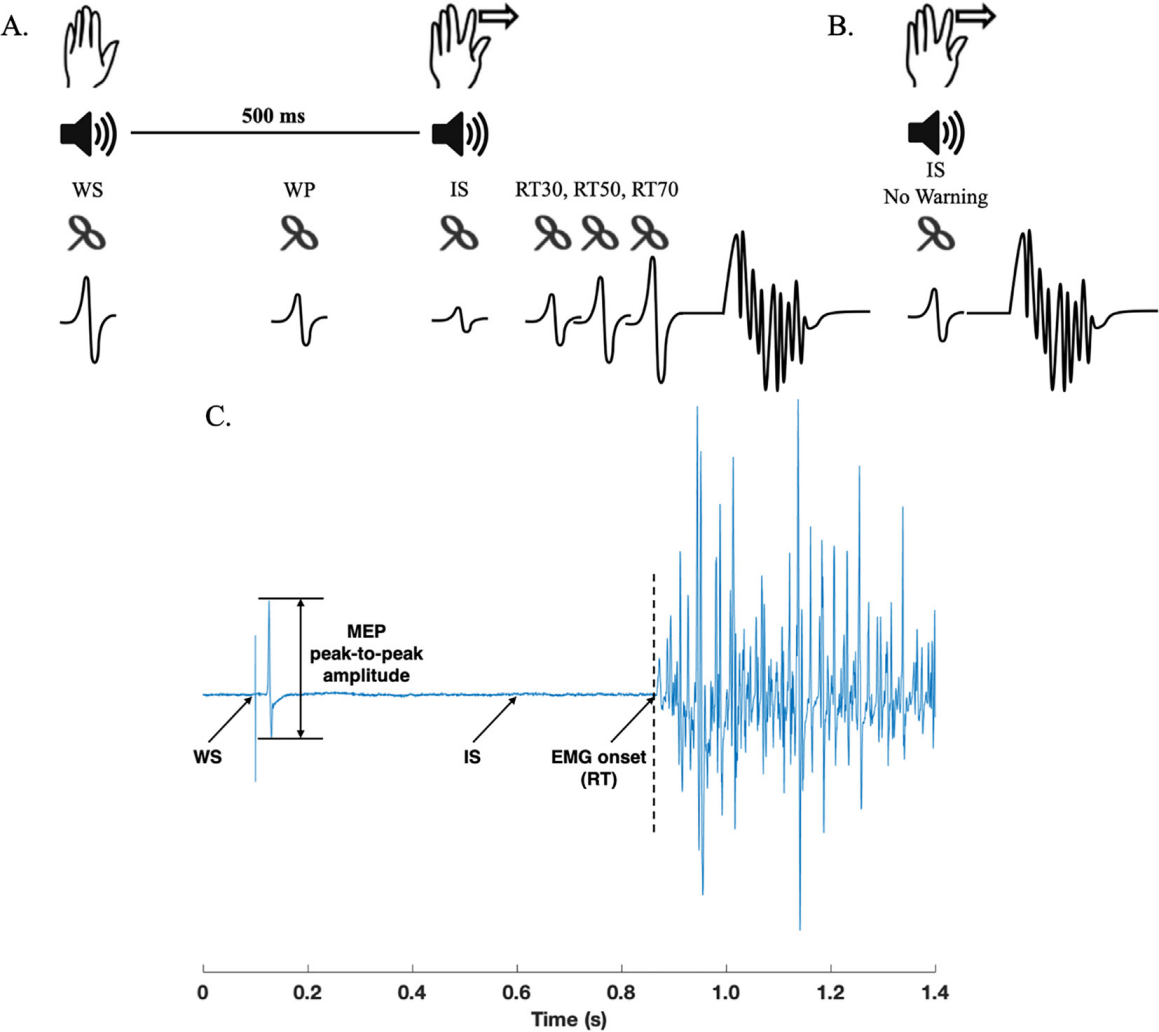
Study design for the simple auditory warned reaction time task (**A**) and the auditory unwarned reaction time task (**B**) used to study the modulation of corticospinal excitability during movement preparation. Warning Stimulus (WS), Warning Period (WP), Imperative Stimulus (IS), Imperative Stimulus (IS) No Warning, 30% RT (RT30), 50% RT (RT50) and 70% RT (RT70) indicate the different time points of stimulation across each task. Participants were instructed to carry out a ballistic index finger abduction after the auditory IS. An example electromyogram (EMG) trace indicating an motor evoked potential (MEP) and reaction time (RT) is shown in panel **C**.

Participants were seated comfortably in a chair facing away from the computer monitor with their hands palm-down on a pillow on their laps and performed a simple warned reaction time task. In each trial of the experiment, an auditory warning stimulus (WS) preceded an auditory imperative stimulus (IS) by a fixed interval of 500 ms. Participants were instructed to respond quickly and accurately to the IS by making a finger abduction using the index finger of the hemiparetic side. Prior to the start of the experiment, participants completed 15 trials of the warned reaction time task without TMS in order to determine their mean baseline reaction time (RT). The main experiment consisted of a single block of 70 trials. To prevent anticipation of the IS and premature responses, catch trials were included where a WS was given with no IS and participants were instructed not to respond on these trials (10 trials). TMS was delivered at six different time points (Fig. 1A): together with the WS (10 trials), late during the foreperiod (WP) defined as 167 ms before the IS (10 trials), together with the IS (10 trials) and at 30%, 50% and 70% of the mean baseline RT (10 trials for each). This resulted in six different TMS conditions, which will be referred to as the following from now on: WS, WP, IS, RT30, RT50, RT70. This allowed us to measure corticospinal excitability during movement preparation. The order of trials was pseudorandomized across the six different TMS timings. Following the completion of 70 trials, a separate single block of 10 trials of unwarned reaction time task was completed. This was not included in the main experiment in order to maintain the effect of the WS. In this task, participants were given an auditory IS with no WS (IS-WS) and were instructed to make a finger abduction using the same finger as previously described as quickly and accurately as possible. TMS was delivered together with the IS (Fig. 1B). In both experiments, stimulus timings were controlled using Signal version 6.04 software connected to a data acquisition system (Power1401, CED, Cambridge, UK). Each trial was 1.5 seconds long and the inter-trial interval was set to 5.5 ± 1.5 seconds.

### 2.5 Fatigue

Fatigue was measured at the very start of the experimental session using the Fatigue Severity Scale (FSS-7), a widely used and validated questionnaire across different conditions ranging from 1 to 7 with an average score of seven being the highest fatigue and a score of one being no fatigue (Johansson et al., 2014).

### 2.6 Data processing and statistical analysis

#### 2.6.1 Screening test scores

Spearman’s Rank Correlations between FSS-7 and a number of measures (age, anxiety, depression, grip strength, NHPT, symbol digit modalities test (SDMT), RMT and RT) were calculated. Wilcoxon rank sum tests were used to assess the difference in FSS-7 across different groups divided based on gender, hemisphere affected and dominant hand being affected. The level of significance was set at p = 0.05 and p-values were adjusted for multiple comparisons using Bonferroni correction.

#### 2.6.2 TMS data

**Fig. 2:**
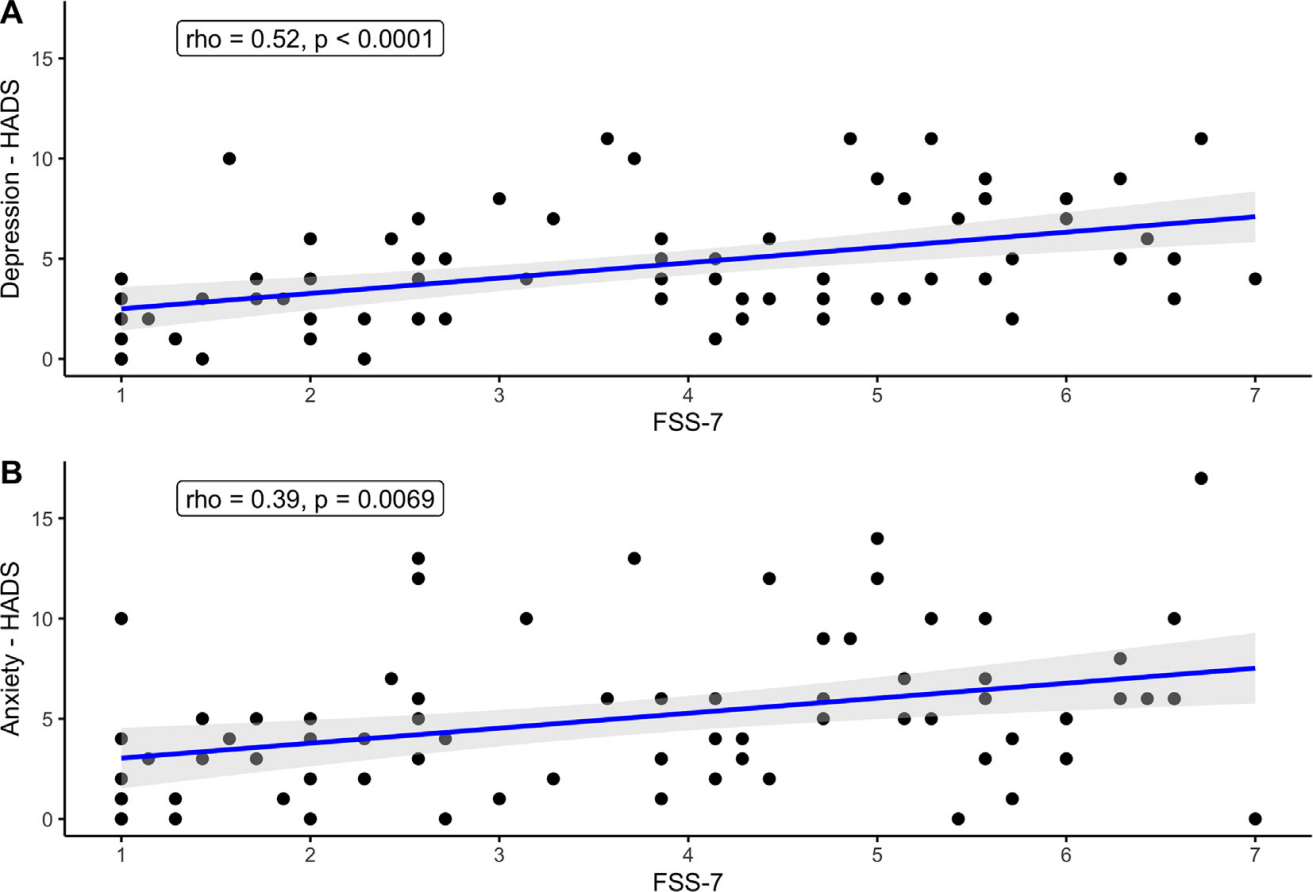
Associations between fatigue as measured by the Fatigue Severity Scale (FSS-7) and (**A**) depression and (**B**) anxiety.

The data files were extracted from Signal into MatLab and were analysed offline using custom-written routines in MatLab (2018a, Mathworks). Two dependent variables were measured on a trial-by-trial basis as follows: (1) MEP peak-to-peak amplitude (Fig. 1C) and (2) reaction time (RT) measured from the time of the IS to the onset of volitional muscle activity (Fig. 1C). Peak-to-peak MEP amplitudes for each condition were estimated from the acquired EMG signal without applying any additional filters. A logarithmic transformation (to the base of e) of single-trial MEP amplitudes was performed before the statistical tests to ensure normality of the samples. Resting EMG was defined as the root mean square (rms) across all trials for each participant in the first 100 ms of each trial (prior to the WS). Thresholds set at five times these levels were used to determine the RT. All trials were then visually inspected and manually corrected to ensure that RT was estimated properly, there was no undue influence of the silent period following stimulation and that no build-up of EMG was apparent before the TMS. Trials in which RT was less than 75 ms or greater than 500 ms were excluded from the final analysis as they represented premature and late responses respectively. Trials were also excluded if the MEP amplitude was less than 0.025 mV. Trials containing outlier MEP amplitudes (Grubb’s test, p < 0.005) were also excluded from the final analysis. On average, 15.4% of TMS trials and 16% of catch trials were excluded across all stroke survivors with a minimum of 7 trials per stimulation condition.

To examine the effect of fatigue on corticospinal excitability and RT, log-transformed MEP amplitudes and RTs were labelled according to the time at which TMS was delivered (WS, WP, IS, RT30, RT50, RT70) and analysed by means of generalized mixed effects models carried out within the R environment for statistical computing (RStudio Version 1.2.5033), using the ‘lme4′ package (Bates et al. 2014). The ‘lmerTest’ package (Kuznetsova et al. 2017) was used to estimate the p-values for the t-test based on the Satterthwaite approximation for degrees of freedom. A stepwise ANOVA based on Satterthwaite’s approximation of degrees of freedom for model selection (lowest AIC value and p-value) was used to identify the combinations of variables that best predicted the outcome variables (MEP amplitude and RT). Based on previous studies we had reason to believe that the change in MEP amplitude over time would follow a quadratic trend whereas RT would follow a linear trend (Bestmann and Duque, 2016, Hannah et al., 2018, Ibáñez et al., 2020). Therefore, we compared the AIC for both the linear and quadratic fit for both MEP amplitude and RT (Table 2). The quadratic model was a better fit for the MEP amplitude data while a linear model was a better fit for the RT data and the effect of Warning. A similar analysis was used to examine the effect of fatigue and condition (Warning condition vs No Warning condition) on corticospinal excitability and RT (Table 2). Assumptions of normality and homoscedasticity of the residuals for each model were assessed visually using quantile-quantile normal plots and fitted- versus residual-value plots. Individual spearman’s rank correlations were carried out between FSS-7 and the dependent variable in each model.

**Table 2:** The result of generalized mixed effects model comparisons across the corticospinal excitability data, the reaction time data and the effect of warning on both corticospinal excitability and reaction time. Participants nested in time are the random effect in each model. Significance levels are indicated by * (* < 0.05). Df = degrees of freedom; AIC = Akaike’s information criterion; Chisq = chi-squared statistic; Chi Df = chi-squared degree of freedom; Pr(>Chisq) = probability value.

Fixed effects	Model Df	AIC	Chisq	Chi Df	Pr(>Chisq)
***Corticospinal Excitability***					
Time	5	6394.4			
Time^2^	6	6334.4	61.6765	1	4.05e−15***
Time^2^ + FSS	7	6334.3	2.4676	1	0.11622
Time^2^ * FSS	9	6331.3	6.9242	2	0.03136*
Time^2^ * FSS + Anxiety + Depression	11	6336.9	0.4249	2	0.80862

***Reaction Time***					
Time	5	−6569.3			
Time^2^	6	−6570.3	2.3481	1	0.12543
Time + FSS	6	−6567.5	0.2015	1	0.65351
Time * FSS	7	−6572.3	6.8826	1	0.008704*
Time * FSS + Anxiety + Depression	11	−6571.8	7.4255	4	0.11504

***Effect of Warning – Corticospinal Excitability***					
Time	6	3009.5			
Time + FSS	7	3008.7	2.7585	1	0.09674
Time * FSS	8	3009.2	1.5054	1	0.21985

***Effect of Warning – Reaction Time***					
Time	6	−2715.8			
Time + FSS	7	−2715.1	1.2095	1	0.2714
Time * FSS	8	−2713.6	0.5360	1	0.4641

For a graphical representation of the results, stroke survivors were divided into two groups, high and low fatigue, based on their FSS-7 scores. A cut-off score of less than four on the FSS-7 was classified as low-fatigue and a score equal or greater than four was classified as high fatigue (Valko et al. 2008). Throughout the analysis, FSS-7 was treated as a continuous scale.

## 3 Results

### 3.1 Demographics

There was a significant positive association between FSS-7 and anxiety (rho = 0.39, p < 0.0069), and depression (rho = 0.52, p < 0.0001), Fig. 2. There was no association between FSS-7 and age, RMT, 0.5 mV intensity and reaction time. There was no difference in FSS-7 in left hemisphere strokes compared to right hemisphere strokes and no difference between males and females.

### 3.2 Corticospinal excitability

**Fig. 3:**
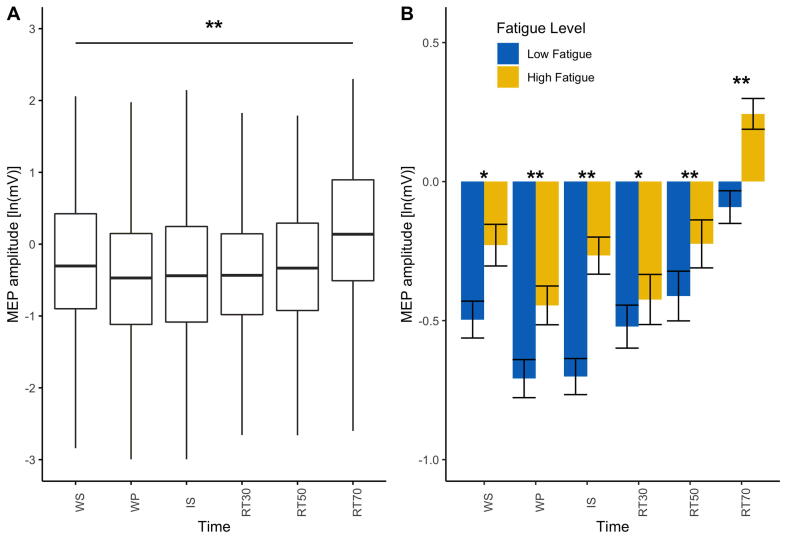
The effect of time and fatigue on motor evoked potential (MEP) amplitude. **A.** Boxplot for MEP amplitude across all stroke survivors for each time point indicating the significant effect of time on MEP amplitude. **B.** Bar plots with standard error bars representing MEP amplitude across all time points with stroke survivors grouped based on their fatigue score indicating the significant interaction between time^2^ and fatigue on MEP amplitude. Fatigue was measured using the Fatigue Severity Scale (FSS-7). Low fatigue patients (FSS-7 < 4) are represented in blue and high fatigue patients (FSS > 4) are represented in yellow. The association between fatigue (FSS-7) and MEP amplitude for each time point is also shown. Significance levels are indicated by * (* < 0.05, ** < 0.001).

A non-linear (quadratic) mixed effects model with time (WS, WP, IS, RT30, RT50 and RT70) and FSS-7 as fixed effects and participant nested in time as random effects best described the rate of change of corticospinal excitability during movement preparation. Including covariates that significantly correlated with FSS-7 (anxiety and depression) did not significantly improve the model. The mixed effects model showed that time^2^ was a significant predictor of MEP amplitude (β = −0.44, t = −4.73, p < 0.001), FSS-7 did not significantly predict MEP amplitude (β = −0.068, t = 0.321, p = 0.75) and the interaction between time^2^ and FSS-7 was a significant predictor of MEP amplitude (β = −0.0066, t = −2.22, p = 0.0263) such that stroke survivors with higher fatigue showed less modulation of corticospinal excitability during movement preparation. There was a significant positive correlation between FSS-7 and MEP amplitude across all time points (WS: rho = 0.1, p = 0.02, WP: rho = 0.13, p = 0.003, IS: rho = 0.21, p < 0.001, RT30: rho = 0.18, p = 0.006, RT50: rho = 0.20, p = 0.003, RT70: rho = 0.13, p = 0.005). All corticospinal excitability data is presented in Fig. 3.

### 3.3 Reaction time

**Fig. 4:**
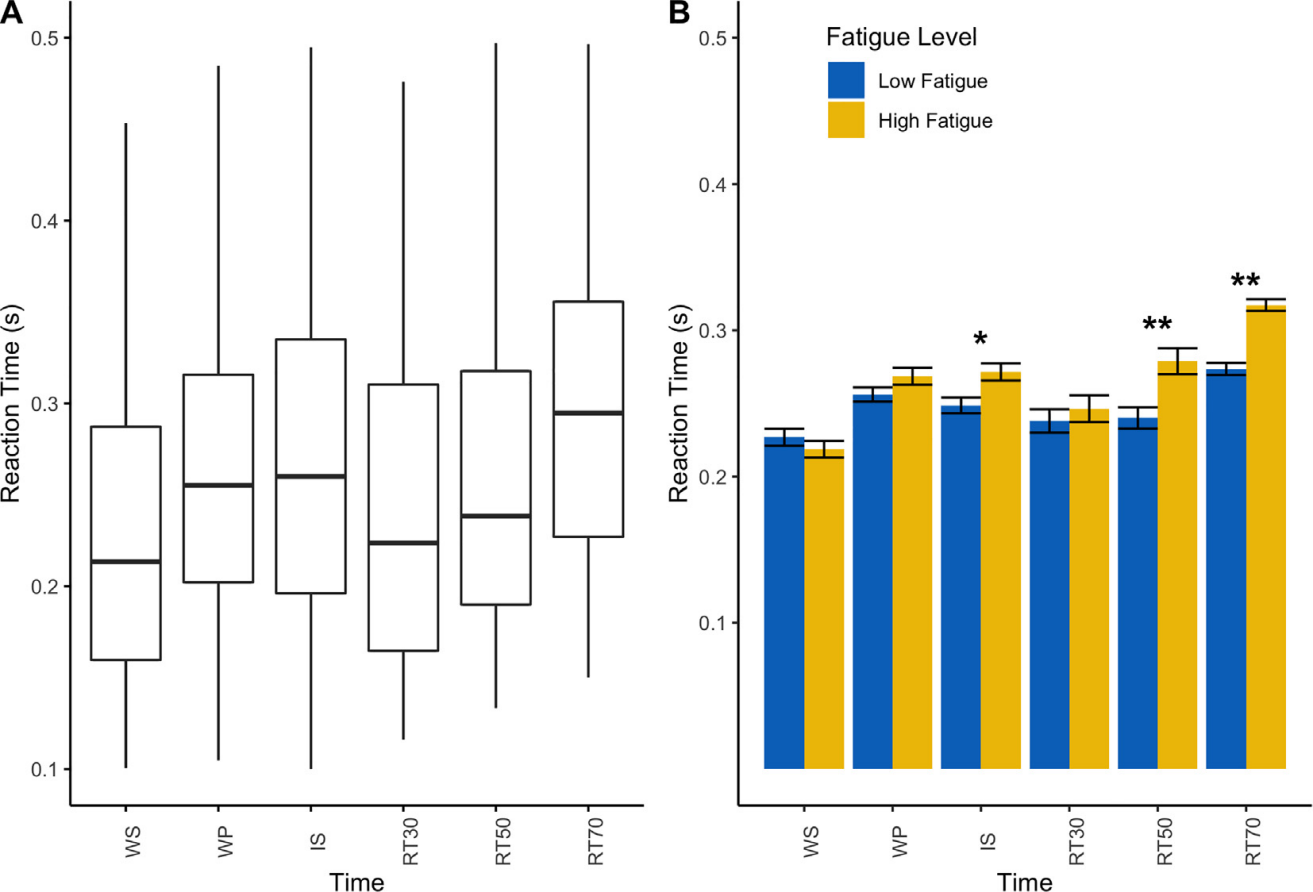
The effect of time and fatigue on reaction time (RT). **A.** Boxplot for RT across all stroke survivors for each time point indicating no significant effect of time on RT. **B.** Bar plots with standard error bars representing RT across all time points with stroke survivors grouped based on their fatigue score indicating the significant interaction between time and fatigue on RT. Fatigue was measured using the Fatigue Severity Scale (FSS-7). Low fatigue patients (FSS-7 < 4) are represented in blue and high fatigue patients (FSS > 4) are represented in yellow. The association between fatigue (FSS-7) and MEP amplitude for each time point is also shown. Significance levels are indicated by * (* < 0.05, ** < 0.001).

A linear mixed effects model with time (WS, WP, IS, RT30, RT50 and RT70) and FSS-7 as fixed effects and participant nested in time as random effects best described the rate of change of reaction time during movement preparation. Including covariates that significantly correlated with FSS-7 (anxiety and depression) did not significantly improve the model. The mixed effects model showed that the fixed effects of time (β = 0.0039, t = 0.980, p = 0.363) and FSS-7 (β = −0.0033, t = −0.854, p = 0.396) did not significantly predict RT. The interaction between time and FSS-7 (β = 0.0024, t = 2.47, p = 0.0159) was a significant predictor of RT such that stroke survivors with higher fatigue showed slower RTs the closer the stimulation time to movement onset. There was a significant positive correlation between FSS-7 and RT only at the IS (rho = 0.1, p = 0.01), RT50 (rho = 0.31, p < 0.001) and RT70 (rho = 0.32, p < 0.001) time points. All RT data is presented in Fig. 4.

### 3.4 Effect of Warning

**Fig. 5:**
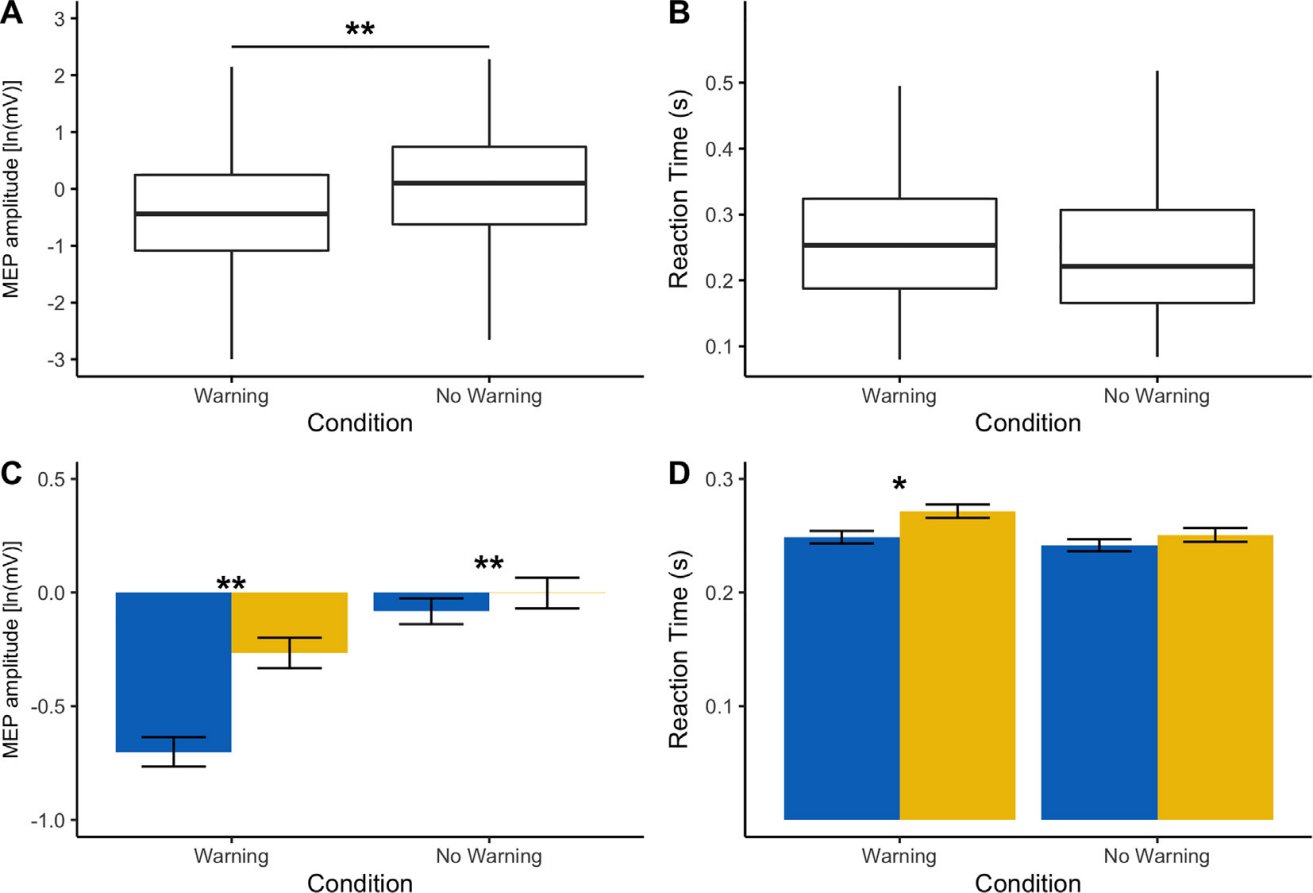
The effect of condition (Warning vs No Warning) and fatigue on motor evoked potential (MEP) amplitude and reaction time (RT). **A, B.** Boxplots for MEP amplitude and RT across all stroke survivors for each condition indicating a significant effect of condition on MEP amplitude but not on RT. **C, D.** Bar plots with standard error bars representing MEP amplitude and RT across both conditions with stroke survivors grouped based on their fatigue score. Fatigue was measured using the Fatigue Severity Scale (FSS-7). Low fatigue patients (FSS-7 < 4) are represented in blue and high fatigue patients (FSS > 4) are represented in yellow. The association between fatigue (FSS-7) and MEP amplitude for each time point is also shown. Significance levels are indicated by * (* < 0.05, ** < 0.001).

A linear mixed effects model with condition (Warning, No Warning) as a fixed effect and participant nested in condition as random effects best described the difference in corticospinal excitability and RT across the two conditions. FSS-7 did not significantly improve the model in either of the two cases. The mixed effects model showed that the fixed effect of condition (β = 0.101, t = 4.072, p < 0.001) was a significant predictor of MEP amplitude across the two conditions (Fig. 5a). The fixed effect of condition (β = −0.004, t = −1.92, p = 0.0592) was not a significant predictor of RT across the two conditions (Fig. 5b). There was a significant positive correlation between FSS-7 and MEP amplitude across both conditions (Warning: rho = 0.21, p < 0.001, No Warning: rho = 0.11, p = 0.004) and a significant positive correlation between FSS-7 and RT in the Warning condition (rho = 0.1, p = 0.001), Fig. 5c-d.

## 4 Discussion

In this study we showed that the modulation of corticospinal excitability during movement preparation changes as a function of fatigue in stroke survivors. Specifically, the higher the level of fatigue, the lower the suppression of corticospinal excitability during movement preparation and the higher the pre-movement facilitation of corticospinal excitability immediately before EMG onset. Reaction times were also greater in stroke survivors with greater fatigue during movement preparation. This was more apparent when TMS was delivered at time point closest to onset of EMG. We also showed that corticospinal excitability is higher in the absence of the warning cue in all stroke survivors. Analysis on the demographics of our patient cohort showed that the higher the levels of anxiety and depression the higher the severity of fatigue.

A number of different models have been put forward to explain the reported modulation in corticospinal excitability during movement preparation from a motor control perspective (Burle et al., 2004, Duque and Ivry, 2009, Duque et al., 2010, Greenhouse et al., 2015, Lebon et al., 2019). Recent studies in nonhuman primates show that neural activity during movement preparation is a necessary component of movement generation and the time spent in the preparatory state can change depending on task demands (Lara et al. 2018). During cue driven movements, such as reaction time tasks where time is available, neuronal activity reaches the preparatory state well before movement onset. The ability to rapidly and consistently reach the correct preparatory state in advance, may allow time to correct small inaccuracies or even speed up movements before the movement itself (Churchland and Shenoy 2007). Similar results have been reported in the mouse motor cortex as well as in humans, where the amount of preparatory inhibition, as measured by pre-movement cortical inhibition, positively correlated with reaction time (Hasegawa et al., 2017, Hannah et al., 2018). Stroke survivors with low levels of fatigue appear to follow a similar pattern of preparatory inhibition as healthy volunteers as has previously been described (Duque et al. 2017). However, with increasing fatigue, there is both reduced preparatory inhibition and earlier pre-movement facilitation before EMG onset. Both peripheral and central components of fatigue have an influence on MEP amplitude (Brasil-Neto et al. 1993). In the current study, any peripheral components of fatigue can be excluded as the cohort of stroke survivors studied were all mildly physically impaired. The differences observed in the modulation of corticospinal excitability as a result of fatigue are primarily driven by central fatigue. Given that the model with the best predictive capacity did not include anxiety and depression scores, suggests that changes seen in motor cortical neurophysiology is exclusively related to fatigue and may be an independent mechanism that drives chronic pathological fatigue.

In the absence of TMS, there is no association between fatigue severity and reaction times. In the TMS conditions however, reaction times are also slower with increasing severity of fatigue. Slower reaction times with greater fatigue during the warned reaction time task maybe due to inability to reach the appropriate preparatory state, indicated by reduced preparatory inhibition, from which to initiate a movement. This could also explain why stroke survivors with high fatigue also have slower self-selected ballistic movement speeds (Kuppuswamy et al. 2015a). Given the undue influence of the stimulation itself on reactions times, particularly at time points close to EMG onset, we were not able to examine the relationship between reaction times and corticospinal excitability in the current experiment. Whether the relationship between reaction times and changes in corticospinal excitability is causal should be explored further. Also, we cannot exclude the possibility that the silent period following stimulation influenced reaction times. The relationship between silent period duration and fatigue has not been explored previously, but given that reaction times were slower with higher severity of fatigue when the stimulation was delivered together with the imperative stimulus, this is unlikely.

A number of studies have attempted to explain the changes reported in corticospinal excitability during movement preparation from a decision making and sensory processing perspective (Klein et al., 2012, Klein-Flügge and Bestmann, 2012, Klein-Flügge et al., 2013, Chiu et al., 2014, Cos et al., 2014, Freeman et al., 2014). MEP amplitudes may be influenced by other non-motor areas such frontal, parietal and subcortical regions that have projections to the pre-motor and motor cortex. Such influences include decision-related variables such as prior probabilities, subjective expected values or sensory evidence, which are computed elsewhere but ultimately influence the state of the motor system. When humans make choices between reaching actions, they tend to choose the one that is less effortful (Cos et al. 2014). This suggests that prior to movement initiation, one can predict the estimated action cost of different movements. Estimated action cost, normally experienced as ‘effort’, can therefore inform both implicit and explicit action choices towards the least effortful one. The estimated action cost of the upcoming movement is inversely proportional to the amplitude of MEPs (Cos et al. 2014). We have previously suggested that PSF is a result of altered perceptual processing, specifically altered perception of effort, associated with actions (Kuppuswamy 2017). The results of the current study, altered modulation of corticospinal excitability, lend support to this hypothesis. The lack of preparatory inhibition and increased pre-movement facilitation may reflect a higher estimated action cost associated with the upcoming movement resulting in the movement being perceived as more effortful. On the contrary, reduced pre-movement facilitation of corticospinal excitability in a visual reaction time task has also been reported in multiple sclerosis fatigue (Morgante et al. 2011). The authors conclude that impairment of areas engaged in motor planning might give rise to fatigue. The contradictory result between the aforementioned study and the current study might be explained by physiological differences previously reported between warned and unwarned reaction time paradigms (Ibáñez et al. 2020).

MEP amplitudes were lower in the warned RT condition compared to the unwarned RT condition irrespective of fatigue severity, while reaction times remained unchanged. Higher MEP amplitude in the unwarned condition is not surprising as the stimulation was given at the time of the imperative stimulus and movement “preparation” was yet to take place, unlike the warned condition. Given the difference in MEP amplitude between the two conditions we would also expect a difference in reaction times. One would expect reaction times to be slower in the unwarned condition when compared to the warned condition. The lack of difference in reaction times across the two conditions could be explained by the inclusion of catch trials in the warned condition experiment, whereas there were no catch trials in the unwarned condition. It has previously been reported that the presence of catch trials slows reaction times (Greenhouse et al. 2015). RTs appear to be longer in the warned condition with increasing severity of fatigue but unchanged in the unwarned condition. This might suggest that stroke survivors with high fatigue are capable of responding as quick as those with low fatigue when time is not available to prepare.

The relationship between RMT of the affected hemisphere and self-reported fatigue may be influenced by other variables than previously suggested (Kuppuswamy et al. 2015b). Corticospinal excitability of the left and right hemispheres behave differently during motor control with asymmetries seen between the two hemispheres (Davidson and Tremblay, 2013, Klein et al., 2016). The asymmetry in corticospinal excitability between the two hemispheres may be driven by driven by altered inter-hemispheric network dynamics that subsequently influence corticospinal excitability (Netz et al., 1995, Ziemann and Hallett, 2001, Ondobaka et al., 2019).

The current study further highlights the overlap of fatigue with other affective symptoms such as anxiety and depression previously described in the literature (De Doncker et al. 2018). Despite the overlap, the mechanisms underlying fatigue appear to be distinct as anxiety and depression scores did not improve the predictive capabilities of the model. This highlights the importance of using a strictly controlled patient cohort when trying to draw conclusions from studies and attempting to develop a mechanistic understanding of affective symptoms.

Despite providing us with useful insights into the mechanisms of post-stroke fatigue, this study is not without limitations. Our findings are limited to the affected hemisphere of non-depressed, mildly impaired stroke survivors. Future studies must investigate corticospinal excitability during movement preparation in a wider stroke population in both the affected and unaffected hemisphere. The high-pass filter settings used in the EMG recording limit our results to the high frequency components of the MEP. As we are interested in modulation of MEP rather than absolute amplitude of MEP (unlike in diagnostic settings as described in (Groppa, 2012)), and with no reason to believe that the low frequency components of MEP will be differentially modulated by fatigue and task, on balance we favoured a 100 Hz high pass filter. There is of course a chance that fatigue and task may differentially affect low frequency components of MEP which is yet to be investigated using lower high-pass filters. The intensity of stimulation to produce a 0.5 mV response was determined at rest. However, when engaged in a task, corticospinal excitability at baseline, despite no EMG activity appears to be different across patients introducing variability into the paradigm. Future studies should ensure that all patients have a similar task-dependent baseline which they can use as a reference to compare the MEP amplitude across different time points. Given the nature of the symptom being investigated, a small number of trials was used at each time point to ensure that all patients could complete the task. We recommend that, given the variability of responses to TMS, future studies should use a block design with sufficient time between blocks to allow patients to rest in order to have a larger number of trials and more robust results. In the current study, we could not make a causal link between corticospinal excitability and reaction time due to the effect of the TMS pulse on reaction time. Using study designs in which reaction time is more closely controlled as demonstrated in a recent experiment (Ibáñez et al. 2020), we might be able to study the effect of pre-movement corticospinal excitability on reaction times.

## 5 Conclusion

The modulation of corticospinal excitability during movement preparation assessed using TMS, changes as a function of fatigue in non-depressed, minimally impaired stroke survivors. Reaction times are also longer when given time to prepare for a movement in those who report high levels of fatigue. Preparatory inhibition, when viewed as a measure of sensory processing of expected stimuli, a reduction in preparatory inhibition in high fatigue may indicate poor sensory processing supporting the sensory attenuation model of fatigue.

## Funding

This work was supported by the Wellcome Trust (202346/Z/16/Z).

## Declaration of Competing Interest

The authors declare that they have no known competing financial interests or personal relationships that could have appeared to influence the work reported in this paper.
